# Increasing the sensitivity of NMR diffusion measurements by paramagnetic longitudinal relaxation enhancement, with application to ribosome–nascent chain complexes

**DOI:** 10.1007/s10858-015-9968-x

**Published:** 2015-08-08

**Authors:** Sammy H. S. Chan, Christopher A. Waudby, Anaïs M. E. Cassaignau, Lisa D. Cabrita, John Christodoulou

**Affiliations:** 1 Institute of Structural and Molecular Biology, University College London and Birkbeck College, London WC1E 6BT, UK

**Keywords:** Diffusion NMR spectroscopy, Paramagnetic longitudinal relaxation enhancement, Ribosome–nascent chain complex, NMR sensitivity enhancement

## Abstract

The translational diffusion of macromolecules can be examined non-invasively by stimulated echo (STE) NMR experiments to accurately determine their molecular sizes. These measurements can be important probes of intermolecular interactions and protein folding and unfolding, and are crucial in monitoring the integrity of large macromolecular assemblies such as ribosome–nascent chain complexes (RNCs). However, NMR studies of these complexes can be severely constrained by their slow tumbling, low solubility (with maximum concentrations of up to 10 μM), and short lifetimes resulting in weak signal, and therefore continuing improvements in experimental sensitivity are essential. Here we explore the use of the paramagnetic longitudinal relaxation enhancement (PLRE) agent NiDO2A on the sensitivity of ^15^N XSTE and SORDID heteronuclear STE experiments, which can be used to monitor the integrity of these unstable complexes. We exploit the dependence of the PLRE effect on the gyromagnetic ratio and electronic relaxation time to accelerate recovery of ^1^H magnetization without adversely affecting storage on N_*z*_ during diffusion delays or introducing significant transverse relaxation line broadening. By applying the longitudinal relaxation-optimized SORDID pulse sequence together with NiDO2A to 70S *Escherichia coli* ribosomes and RNCs, NMR diffusion sensitivity enhancements of up to 4.5-fold relative to XSTE are achieved, alongside ~1.9-fold improvements in two-dimensional NMR sensitivity, without compromising the sample integrity. We anticipate these results will significantly advance the use of NMR to probe dynamic regions of ribosomes and other large, unstable macromolecular assemblies.

## Introduction

NMR diffusion measurements are a powerful probe of biomolecular structure and dynamics in which the translational properties of molecules can be examined non-invasively, using a very wide variety of gradient echo NMR experiments (Johnson 1999). These measurements can be used to determine diffusion coefficients, which in turn can be related to hydrodynamic radii and hence molecular structure by the Stokes–Einstein equation. The development of NMR diffusion methods has thereby advanced studies in a wide range of areas in biology, such as the analysis of peptide aggregation and amyloid formation (Baldwin et al. 2008); macromolecular crowding effects (Li et al. 2009); protein–ligand binding events (Lucas and Larive 2004); and in-cell NMR to distinguish between intra- and extracellular proteins (Waudby et al. 2012). Furthermore, NMR measurements of diffusion have been used to investigate how secondary structure and hydrophobic clusters affect the hydrodynamic radii within different conformational ensembles including partially folded and molten globule states (Wilkins et al. 1999). With increasing applications of NMR spectroscopy in understanding the biology of complex systems, NMR diffusion measurements are likely to develop growing prominence.

The measurement of translational diffusion has also played an important role in NMR studies of large macromolecular assemblies, including investigations of ribosomal particles (Christodoulou et al. 2004; Cabrita et al. 2009; Hsu et al. 2007; Eichmann et al. 2010). The study of such complexes is of major biological interest, but the high molecular weight and the resulting low maximum achievable concentrations, most often combined with limited sample lifetimes, commonly results in very weak signals that present significant spectroscopic challenges (Waudby et al. 2013). An example of this is seen in recent studies of ribosome–bound nascent chain complexes (RNCs), in which sample lifetimes are limited primarily by release of the nascent chain from the ribosome before degradation of the ribosome itself (Waudby et al. 2013). The continuous monitoring of translational diffusion is therefore essential to ensure that the observed resonances arise from an intact complex. In particular, isotope-edited diffusion experiments, and especially the heteronuclear stimulated-echo (XSTE) experiment (Ferrage et al. 2003) have been critical in allowing the attachment of the isotopically-labelled nascent chain to the (unlabeled) ribosome to be monitored specifically (Cabrita et al. 2009; Hsu et al. 2007; Eichmann et al. 2010; Waudby et al. 2013), an approach similar to one first used to study the dynamic regions of free ribosomes (Christodoulou et al. 2004).

Given such constraints to NMR studies of ribosomal particles, continual improvements in experimental sensitivity are central to progress in this field. Large gains in sensitivity and resolution have been made through the availability of high-field spectrometers (Rovnyak et al. 2004) and cryogenic probes (Kovacs et al. 2005). Transverse relaxation optimized spectroscopy (TROSY) (Pervushin et al. 1997; Fernández and Wider 2003) and methyl-TROSY (Tugarinov et al. 2003) in combination with advanced isotopic labeling schemes (Tugarinov et al. 2006) have revolutionised the study of large systems by NMR spectroscopy, such as the 900 kDa GroEL–GroES complex (Fiaux et al. 2002) and 670 kDa 20S proteasome (Sprangers and Kay 2007), and these methods are beginning to be applied to the study of RNCs (Eichmann et al. 2010). Furthermore, other techniques such as non-uniform (sparse) sampling (Hyberts et al. 2012) or non-uniform weighted sampling (Waudby and Christodoulou 2012) may also further contribute to sensitivity improvements in multi-dimensional NMR by sampling more efficiently on the Nyquist grid.

In typical NMR measurements, the majority of spectrometer time (>90 %) is devoted to the inter-scan recovery delay, during which no new data is actively acquired but in which the sample magnetization slowly recovers towards Boltzmann equilibrium through the process of longitudinal relaxation. The impact of this delay on experimental sensitivity has been considered since the earliest days of Fourier transform NMR, when optimal signal recovery was found to be intrinsically linked to the length of the recovery delay, as well as the excitation flip angle (Ernst and Anderson 1966). More recently, longitudinal cross-relaxation following selective excitation of a subset of spins has been exploited to accelerate the longitudinal relaxation of observed spins. This optimization of longitudinal relaxation was first applied in LTROSY experiments (Pervushin et al. 2002), and later coupled with selective Ernst angle excitation and fast repetition rates in the SOFAST-HMQC experiment (Schanda et al. 2005). Since saturation of water protons is avoided, the latter experiment has proven to be particularly advantageous for intrinsically disordered proteins (IDPs) as there is an additional transfer of ‘cold’ water protons onto the protein via rapid amide proton exchange (Gil et al. 2013).

Longitudinal relaxation-optimized experiments have also been developed for protein diffusion measurements. The XSTE scheme was the first pulse sequence to exploit the disparities in longitudinal relaxation times between different nuclei, storing the spatially-encoded magnetization on ^15^N spins rather than on ^1^H (as in homonuclear stimulated echoes) to reduce the loss of signal during the diffusion delay (Ferrage et al. 2003). This method was later combined with the selective excitation of amide protons (BEST-XSTE), and Ernst angle excitation with shorter recovery times (SOFAST-XSTE) (Augustyniak et al. 2011) to increase experimental sensitivity. More recently, the SORDID experiment has been proposed, which provides further signal gains by allowing the recovery of ^1^H magnetization to occur simultaneously with the diffusion of spatially-encoded ^15^N longitudinal magnetization, thereby reducing the overall experimental time to approximately half that of XSTE-type experiments (Augustyniak et al. 2012).

An orthogonal approach to accelerate longitudinal relaxation is the introduction of soluble paramagnetic compounds into NMR samples (Eletsky et al. 2003; Hiller et al. 2005; Wickramasinghe et al. 2007; Cai et al. 2006). The solvent paramagnetic relaxation enhancement (PRE) effect, which arises from long-range magnetic dipolar interactions between unpaired electrons from the paramagnetic center and a nucleus, results in an increase in both longitudinal and transverse relaxation rates (Otting 2010):

(1) $$R_{1}=R_{1\text{d}}+R_{1\text{p}}$$

(2) $$R_{2}=R_{2\text{d}}+R_{2\text{p}}$$

where *R*_1d_ and *R*_2d_ are the diamagnetic relaxation rates in the absence of a PRE agent, and *R*_1p_ and *R*_2p_ are the additional paramagnetic contributions to relaxation. In the absence of specific interactions between the protein and the PRE agent, paramagnetic relaxation can be attributed to fluctuations in the electron–nuclear dipolar interaction that arises from a combination of intrinsic electron spin relaxation and the translational diffusion of the PRE agent relative to the nucleus (Bertini et al. 2001; Bernini et al. 2009). In this ‘outer sphere’ or diffusional model, the paramagnetic components of the relaxation rates are given by (Helm 2006):

(3) $$R_{1\text{p}}=\frac{32\pi }{405}{\left(\frac{\mu_{0}}{4\pi }\right)}^{2}\frac{1000N_{\text{A}}[\text{M}]\gamma_{\text{n}}^{2}\gamma_{\text{e}}^{2}\hbar^{2}S(S+1)}{d(D_{\text{M}}+D_{\text{P}})}\times [7J_{2}(\omega_{\text{e}})+3J_{1}(\omega_{\text{n}})]$$

(4) $$R_{2\text{p}}=\frac{16\pi }{405}{\left(\frac{\mu_{0}}{4\pi }\right)}^{2}\frac{1000N_{\text{A}}[\text{M}]\gamma_{\text{n}}^{2}\gamma_{\text{e}}^{2}\hbar^{2}S(S+1)}{d(D_{\text{M}}+D_{\text{P}})}\times \left[4J_{1}(0)+13J_{2}(\omega_{\text{e}})+3J_{1}(\omega_{\text{n}})\right]$$

where *μ*_0_ is the permeability of free space, *N_A_* is Avogadro’s constant, [M] is the concentration of the paramagnetic species, *γ*_n_ and *γ*_e_ are the nuclear and electronic gyromagnetic ratios, *ω*_n_ or *ω*_e_ are the nuclear and electronic Larmor frequencies, *ħ* is the reduced Planck constant, *S* is the electron spin quantum number, *D*_M_ and *D*_P_ are the diffusion coefficients of the paramagnetic species and the protein, and *d* is the distance of closest approach between the paramagnetic center and nuclear spin. The spectral density functions *J_i_*(*ω*) (with *n* = 1, 2) are:

(5) $$J_{n}(\omega )=\frac{1+\frac{1}{4}{\left(n\omega \tau_{\text{D}}+\frac{\tau_{\text{D}}}{T_{ne}}\right)}^{1/2}}{1+{\left(n\omega \tau_{\text{D}}+\frac{\tau_{\text{D}}}{T_{ne}}\right)}^{1/2}+\frac{4}{9}\left(n\omega \tau_{\text{D}}+\frac{\tau_{\text{D}}}{T_{ne}}\right)+\frac{1}{9}{\left(n\omega \tau_{\text{D}}+\frac{\tau_{\text{D}}}{T_{ne}}\right)}^{3/2}}$$

where *T*_1*e*_ and *T*_2*e*_ are the longitudinal and transverse electron relaxation times, and the diffusional correlation time *τ*_D_ = *d*^2^/(*D*_M_ + *D*_P_) (Bertini et al. 2001). For small paramagnetic compounds, *τ*_D_ is on the order of 2 ns (based on a closest approach distance of 1 nm and a hydrodynamic radius of 5 Å). In contrast, their electronic relaxation times can vary over several orders of magnitude, from ps to μs (Bertini et al. 2001), and this can strongly influence the relative magnitudes of *R*_1p_ and *R*_2p_.

A variety of paramagnetic relaxation agents have been employed to enhance the sensitivity of NMR experiments. For example, in solid-state NMR, where longitudinal relaxation is particularly slow, sensitivity enhancements of up to 2.9-fold were obtained in the presence of a Cu^(II)^–EDTA complex (Wickramasinghe et al. 2007), while in the solution-state, doping samples with 1 mM Gd^(III)^–DO2A^–^ provided sensitivity increases of up to 1.28-fold in 2D TROSY experiments of the 800 kDa chaperone GroEL (Hiller et al. 2005). However, in both cases the acceleration of ^1^H longitudinal relaxation was accompanied by increased line broadening effects associated with significantly greater increases in the transverse relaxation rates; this can be attributed to the long electron spin relaxation times of Cu^(II)^ and Gd^(III)^ (10^–9^–10^–8^ s) (Bertini et al. 2001) which are comparable to the diffusional correlation time. While such transverse PRE effects can be useful as structural probes of solvent accessibility (Clore and Iwahara 2009; Madl et al. 2011), in general these effects are deleterious for optimizing the sensitivity of experiments.

In contrast, paramagnetic metal ions such as Fe^(III)^ and Ni^(II)^ with electronic relaxation times much shorter than their diffusional correlation time (e.g. ~10^–11^ s for Ni^(II)^) (Rantaharju et al. 2014) can be used to reduce recycle times by accelerating proton longitudinal relaxation rates with only very marginal effect on transverse relaxation (Cai et al. 2006; Theillet et al. 2011). In this case, Eqs. 3 and 4 reach a limit in which the relaxation rate *R*_2p_ ≈ 1.2 *R*_1p_. In fact, this limit is not sensitive to the details of the relaxation mechanism, and an ‘inner sphere’ model in which the paramagnetic agent binds transiently to the protein was previously analyzed and found to give similar results (Cai et al. 2006). Since longitudinal relaxation in biological molecules is typically much slower than transverse relaxation (*R*_1*d*_ < *R*_2*d*_), *R*_1_ can therefore be increased significantly with little relative effect on *R*_2_ (Eqs. 1, 2); we term this the paramagnetic longitudinal relaxation enhancement (PLRE) effect.

In this study, we exploit the dependence of the PLRE effect of NiDO2A (a neutral and hydrophilic chelate of Ni^(II)^) on the squared gyromagnetic ratio (Eqs. 3, 4) to enhance the sensitivity of heteronuclear STE diffusion measurements, where magnetization spatially encoded on ^1^H is stored longitudinally on ^15^N spins, which has a smaller gyromagnetic ratio (Ferrage et al. 2003). We demonstrate that further synergistic sensitivity enhancements can be achieved by using longitudinal relaxation-optimized SORDID experiments to exploit shortened recycle times in NiDO2A-doped samples of both α-synuclein, an intrinsically disordered protein (IDP), and ddFLN5, a globular immunoglobulin domain from *Dictyostelium discoideum* (ddFLN) that we have previously used in co-translational folding studies (Cabrita et al. 2009; Hsu et al. 2009). Crucially, when applied to isotopically labeled *Escherichia coli* 70S ribosomes, and to a ribosome–nascent chain complex (Cabrita et al. 2009; Hsu et al. 2007) we show that similarly large gains in sensitivity can be achieved by this method without compromising sample integrity. We therefore expect these enhancements will greatly facilitate future NMR investigations of such large, dilute, and unstable macromolecular machines.

## Experimental section

### Preparation and biochemical evaluation of uniformly ^15^N-labelled proteins, ribosomes and RNCs

Established protocols were used for the production and purification of uniformly ^15^N-labelled α-synuclein (Waudby et al. 2010) and ddFLN5 (Hsu et al. 2009) from *E. coli* BL21 (DE3) Gold cells (Stratagene). Intact and uniformly ^15^N-labelled 70S ribosomes were isolated from *E. coli* as previously described (Christodoulou et al. 2004). The RNC used in this study is a modification of the previously described ddFLN_646–839_ construct (Cabrita et al. 2009). Here, the fifth immunoglobulin domain of ddFLN (ddFLN5) is linked to a 31-residue sequence derived from the sixth filamin domain of ddFLN and the SecM stalling motif (Cabrita et al. in preparation). The RNC stability and integrity was monitored over time, by collecting aliquots of a sample incubated in parallel to NMR diffusion experiments (as described below) and evaluated by observing the presence of the tRNA-bound nascent chain by western blot in which the samples are run on SDS-PAGE under low pH conditions (Cabrita et al. in preparation); the nascent polypeptide was detected using both anti-His (Qiagen) and anti-SecM antibodies (a kind gift from Bernd Bukau, University of Heidelberg, Germany).

### Preparation of NiDO2A

DO2A [1,4,7,10-tetraazacyclododecane-1,7-bis(acetic acid)] was purchased from Macrocyclics, Inc. (Dallas, Texas, USA) as a lyophilized salt (H_2_DO2A.4HCl). A 5 % molar excess of DO2A (200 mg) was mixed with anhydrous nickel (II) chloride (57 mg) (Sigma-Aldrich UK), and dissolved in 5 mL of deionized water. The solution was adjusted to neutral pH, coinciding with a colour change from blue to purple, and allowed to stand overnight at room temperature (Cai et al. 2006). Salt and excess DO2A were removed by Dowex Retardion 11A8 ion-exchange resin (Sigma-Aldrich UK) packed into a column and connected to an ÄKTA FPLC system. The absorption at wavelength 545 nm and the conductivity were monitored for the elution of NiDO2A and excess salt, respectively. Desalting followed by lyophilisation and redissolving of the sample was repeated two to three times, and again immediately before use in NMR experiments. The concentration of NiDO2A stock solution (determined by measuring dry mass of NiDO2A before dissolving) was adjusted to a final concentration of 0.5–1.0 M and added to NMR samples as required. The extinction coefficient of NiDO2A at 545 nm was determined as ε = (7.51 ± 0.36) M^–1^ cm^–1^.

### NMR spectroscopy

NMR samples of α-synuclein (200 μM) were prepared in 40 mM Na_2_HPO_4_ and 150 mM NaCl, pH 6.6 in 10 % (v/v) D_2_O and 0.001 % (w/v) DSS. NMR samples of ddFLN5 (100 μM), 70S ribosomes (10 μM) and RNCs (10 μM) were prepared in Tico buffer [10 mM HEPES, 30 mM NH_4_Cl, 12 mM MgCl_2_, 1 mM EDTA, 1 mM BME, pH 7.5, in 10 % (v/v) D_2_O] with protease inhibitors (Sigma-Aldrich UK) and 0.001 % (w/v) DSS. All NMR experiments were recorded using 5 mm diameter Shigemi tubes, as the reduced sample height inhibits the onset of convection (Chung et al. 1970). NMR data for α-synuclein and ddFLN5 were acquired at 283 and 298 K respectively, on a 500 MHz Bruker Avance III spectrometer equipped with a TXI room temperature probe. NMR data for ribosomes and RNCs, prepared in Tico buffer, were acquired at 298 K on a 700 MHz Bruker Avance III spectrometer equipped with a TXI cryoprobe. Both spectrometers were equipped with unidirectional gradient coils generating maximum gradient strengths of 0.55 T m^–1^.

Two-dimensional ^1^H–^15^N SOFAST-HMQC spectra (Schanda et al. 2005) were acquired with 16 (α-synuclein and ddFLN5), 32 (ribosomes), or 256 (RNC) scans; 128 complex points and sweep widths of 23 (α-synuclein), 33 (ddFLN), or 32 ppm (ribosomes and RNC) in the indirect ^15^N dimension; and 1024 points and sweep widths of 20 (α-synuclein and ddFLN5), 14 (ribosomes), or 16 ppm (RNC) in the direct (^1^H) dimension, corresponding to acquisition times of ca. 50 ms. 2D spectra were recorded with a 50 ms inter-scan recovery delay. Using the same parameters, series of one-dimensional ^1^H–^15^N SOFAST HMQC spectra were also acquired as pseudo-2D experiments, with recovery times varying from 50 ms to 1 s. Spectra were referenced to DSS (Wishart et al. 1995) and processed with nmrPipe (Delaglio et al. 1995) using cosine-squared window functions. Exponential window functions were used in the direct dimension for ribosome and RNC spectra.

^1^H longitudinal and transverse relaxation rates were measured using ^1^H–^15^N HSQC experiments incorporating initial inversion-recovery and spin-echo elements respectively. Effective longitudinal relaxation rates were measured following both hard and amide-selective square (400–560 ms) inversion pulses. ^15^N longitudinal and transverse relaxation rates were measured using standard Bruker library sequences. In all cases, experiments were acquired as pseudo-2D experiments, and 1D integrals of the amide region were fitted to determine approximate relaxation rates averaged over all residues in the protein.

^15^N-XSTE and SORDID diffusion experiments were acquired with diffusion delays Δ varied between 110 and 350 ms. The gradient strength *G*was varied(between0.28and 0.53 T m^–1^) to obtain a constant echo attenuation *I/I*_0_ in all experiments, by maintaining a constant value of the product *G*^2^(Δ – *δ*/3 – *τ*/2) ≈ *G*^2^Δ according to the Stejskal–Tanner equation (Stejskal and Tanner 1964; Wu et al. 1995):

(6) II0=exp[−Dγ2σ2G2δ2(Δ−δ3−τ2)]

where *D* is the diffusion coefficient, *γ* is the gyromagnetic ratio, *δ* is the length of the encoding and decoding gradient pulses (*δ* = 4 ms), *σ* is the shape factor of the gradient pulses (*σ* = 0.9 for the trapezoidal gradient shapes used in this work), and *τ* is the delay between the bipolar gradient pulses. In order to avoid damage to the probe due to the high rate of repetition in SORDID experiments, the time-averaged power in the gradient coil, $\bar{P}\propto \frac{1}{T_{scan}}\int_{0}^{T_{scan}}I{\left(t\right)}^{2}\text{d}t$, where current *I* is directly proportional to the applied gradient strength *G*, was considered and limited according to the probe specification. For the RNC, we sought to compare constant echo attenuation between XSTE with Δ = 100 ms (*G* = 5, 95 % *G*_max_) and, to meet the limits of $\bar{P}$, a longer diffusion delay of Δ = 190 ms was used for SORDID experiments (*G* = 10.4, 69.5 % *G*_max_). XSTE spectra were acquired with 64 scans, 1024 complex points and sweep width of 20 (α-synuclein and ddFLN5) or 15 ppm (ribosomes and RNC) in the ^1^H dimension, a recovery delay of 1 s, and acquisition times of 51.25 ms (α-synuclein and ddFLN5) or 48.79 ms (ribosomes and RNC). SORDID experiments were recorded using 256 (α-synuclein and ddFLN5), 64 (ribosomes), or 480 scans (RNC), 1024 complex points and sweep width of 20 (α-synuclein and ddFLN5) or 15 ppm (ribosomes and RNC) in the ^1^H dimension, and acquisition times of 51.25 ms (α-synuclein and ddFLN5) or 48.79 ms (ribosomes and RNC). By considering the effect of overlapping scans, additional phase cycling was introduced in the SORDID experiment to select heteronuclear coherences and improve solvent suppression (detailed in Fig. S1). Diffusion spectra were processed with nmrPipe (Delaglio et al. 1995) using cosine-squared window functions, or an exponential window function for ribosome and RNC samples, linear baseline correction and solvent suppression filters.

### NMR data analysis

Processed 2D spectra were analyzed in CCPN Analysis (Vranken et al. 2005). Diffusion spectra were imported into MATLAB (R2014b, The MathWorks Inc.), and following integration of amide regions diffusion coeficients *D* were calculated using the Stejskal–Tanner equation (Eq. 6). The standard deviation of the noise integral was calculated using all diffusion spectra from each sample. The experimental sensitivity (signal-to-noise ratio per unit time, SNR_*t*_), was calculated as:

(7) $${\text{SNR}}_{t}=\frac{\text{total}\;\text{amide}\;\text{envelope}\;\text{integral}}{\text{standard}\;\text{deviation}\;\text{of}\;\text{noise}\;\text{integral}}\times \frac{1}{\sqrt{N_{\text{scan}}T_{\text{scan}}}}$$

where *N*_scan_ is the number of scans and *T*_scan_ is the total time for one scan. The values for experimental sensitivity were subsequently normalized according to the maximum SNR_*t*_ obtained for XSTE experiments in the absence of NiDO2A. The resulting SNR_t_ data points, plotted over varying Δ, were fitted to their theoretical sensitivities derived by detailed analysis of the trajectory of magnetization during the pulse sequences:

(8) $${\text{SNR}}_{t,\text{XSTE}}=\frac{A\;\text{exp}\left(-4R_{2}^{\text{H}}\tau \right)\;\exp\left(-4R_{2}^{\text{N}}\tau \right)\text{exp}\left(-R_{1}^{\text{N}}T_{\text{N}}\right)}{\sqrt{N_{\text{scan}}T_{\text{scan}}}}\times \left[1-\text{exp}\left(-T_{\text{rec}}R_{1}^{\text{H}}\right)\right]$$

(9) $${\text{SNR}}_{t,\;\text{SORDID}}=\frac{2A\;\text{exp}\left(-4R_{2}^{\text{H}}\tau \right)\text{exp}\left(-4R_{2}^{\text{N}}\tau \right)\text{exp}\left[-2\delta^{\prime }(-R_{1}^{\text{N}}+R_{1}^{\text{H}})\right]\text{exp}\left[-(\Delta -2\delta^{\prime }-6\tau )R_{1}^{\text{N}}\right]}{\sqrt{2N_{\text{scan}}T_{\text{scan}}}}\left[1-\text{exp}\left(-T_{\text{rec}}R_{1}^{\text{H}}\right)\right]$$

where *A* is a scaling factor, *T*_N_ is the length of period during which magnetization is stored on ^15^N nuclei, *T*_rec_ is the recovery time, *τ* is the delay for INEPT transfer (*τ* = |1/4*J*_NH_| = 2.72 ms, for *J*_NH_ ≈ –92 Hz), and *δ′* is the length of the delay as described in the pulse sequence (Fig. S1). An additional factor of 2 is included in the theoretical SORDID sensitivity to account for each diffusion delay extending over two scans. The measured transverse relaxation rates $R_{2}^{H}$ and $R_{2}^{\text{N}}$ were directly inputted into the fit (as their exponential factors only attenuate the scaling), and a global fitting was used to determine the individual longitudinal relaxation rates $R_{1}^{\text{H}}$ and $R_{1}^{\text{N}}$ of each sample, and the shared amplitude A between each diffusion experiment. Errors were calculated by bootstrapping of residuals (Efron et al. 1994).

## Results

**Fig. 1:**
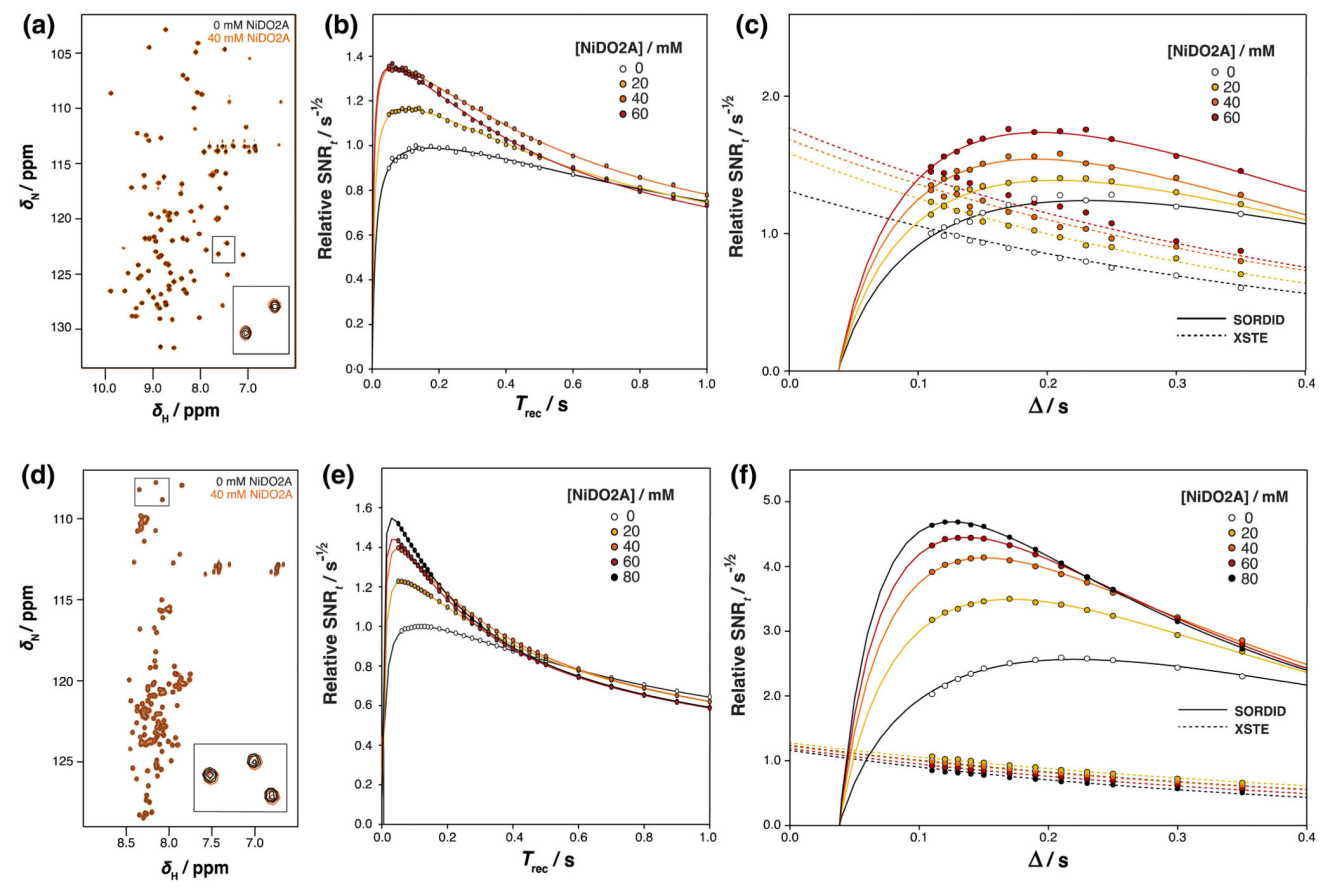
The effect of paramagnetic longitudinal relaxation enhancement on the sensitivity of NMR measurements of **a–c** ddFLN5 and **d–f** α-synuclein. Overlay of 2D ^1^H–^15^N SOFAST-HMQC spectra **a, d** of isolated proteins in the presence and absence of 40 mM NiDO2; insets show magnified views of highlighted resonances. The relative sensitivity of **b, e** 1D ^1^H–^15^N SOFAST-HMQC experiments as a function of the recovery delay *T*_rec_, and of **c, f** XSTE and SORDID diffusion experiments as a function of the diffusion delay Δ. Data are fitted to their theoretical sensitivity expressions (Eqs. 8, 9)

We initially explored the effect of NiDO2A on the sensitivity of the 2D SOFAST-HMQC, and XSTE and SORDID NMR diffusion measurements of two well-characterized isolated proteins. 2D ^1^H–^15^N SOFAST-HMQC experiments were acquired on uniformly ^15^N-labeled samples of α-synuclein (Fig. 1a) and ddFLN5 (Fig. 1d), in both the absence and presence of 40 mM NiDO2A PLRE agent. As seen with previous studies (Cai et al. 2006; Theillet et al. 2011), NiDO2A did not induce chemical shift changes and only marginal line broadening was observed. This observed absence of interaction has been attributed to the very tight affinity of Ni^2+^ to DO2A^2–^ ligand [sub-femtomolar *K*_d_ (Chang et al. 1999)], which outcompetes the interaction of Ni^2+^ with the hexahistidine purification tag present in ddFLN5. Furthermore, the overall neutral charge of the resulting complex is thought to minimize electrostatic interactions with nucleic acids and proteins (Cai et al. 2006).

To quantify the SOFAST-HMQC sensitivity enhancements due to NiDO2A, a series of 1D ^1^H–^15^N SOFAST-HMQC experiments were acquired with the recovery delay varied between 50 ms and 1 s, and the SNR of the resulting spectra was calculated by integration of the amide envelope. For ddFLN5, the sensitivity improved up to 1.4-fold as the NiDO2A concentration was increased to 40 mM NiDO2A (Fig. 1b), and under these conditions 2D SOFAST-HMQC experiments (Fig. 1a) showed residue-specific sensitivity enhancements of 1.45 ± 0.17 (SD) distributed uniformly across the entire protein sequence (Fig. S2). However, in the presence of the highest NiDO2A concentration tested (60 mM) a smaller improvement in sensitivity was observed (1D SOFAST-HMQC sensitivity increase of 1.3-fold and mean residue-specific increase of 1.25 ± 0.17) due to increasing ^1^H transverse relaxation rates. Nevertheless, line widths did not broaden such that resonances became overlapped, and for NiDO2A concentrations up to 60 mM well-resolved spectra could still be acquired.

We next explored the use of NiDO2A with isolated ddFLN5 in NMR diffusion experiments (Fig. 1c). We compared the sensitivity of the XSTE experiment (Ferrage et al. 2003) with the longitudinal relaxation-optimized SORDID experiment (Augustyniak et al. 2012), adjusting *G*_max_ to obtain constant echo attenuation across a range of diffusion delays between 110 and 350 ms. The data were fitted globally using a constant scaling factor and experimentally determined transverse relaxation rates to theoretical expressions for SNR_*t*_ (Eqs. 8, 9) obtained by analysis of the trajectory of magnetization during the XSTE and SORDID pulse sequences (Table 2). Good agreement was generally found between the fitted ^15^N and ^1^H longitudinal relaxation rates and those measured directly using inversion-recovery experiments (Table 1). Where differences arise, we suggest this may in part be due to the differential weighting of different residues in the one-dimensional amide envelope observed by diffusion NMR and relaxation measurements. Advantageously to heteronuclear diffusion NMR measurements, only small increases in $R_{1}^{\text{N}}$ were observed as expected (Eq. 3), such that loss of sensitivity is minimized during storage of magnetization on ^15^N nuclei.

We found that sensitivity was increased by between 1.0-and 1.9-fold, depending on the diffusion delay Δ, when using SORDID in place of the XSTE experiment in the absence of NiDO2A (Fig. 1c). The greatest enhancements were observed for long diffusion delays, which may be attributed to the slow ^1^H longitudinal relaxation rate observed for ddFLN5 (Table 1). This favours the long recovery time of 1 s provided in XSTE experiments, but not in SORDID experiments where recovery times are shorter than (and coupled to) the diffusion delay. As sensitivity is limited in this case mainly by the slow $R_{1}^{\text{H}}$, the use of PLRE agents is clearly advantageous, and we observed sensitivity increases of ~50 % for both XSTE and SORDID experiments in the presence of 20–60 mM NiDO2A.

We found that ^1^H longitudinal relaxation accelerates with increasing NiDO2A concentrations both in our fitted and observed rates (Table 1). However, as the XSTE employs a fixed recovery delay then once a sufficiently fast $R_{1}^{\text{H}}$ is achieved (where magnetization fully returns to equilibrium within the recovery delay) then the signal is attenuated to a greater extent by fast transverse relaxation rates. This is reflected in the large uncertainties of the fitted $R_{1}^{\text{H}}$ rates for XSTE (Table 1). However, those fitted to SORDID measurements were found to be systematically lower than those measured experimentally following a soft inversion pulse. This may reflect the several additional hard pulses in the SORDID sequence during longitudinal relaxation recovery, such that the overall excitation of spins is expected to be less selective than the single selective inversion pulse that was used to measure $R_{1}^{\text{H}}$ directly. Overall, the combined use of SORDID and 60 mM NiDO2A for ddFLN5, with optimization of Δ, provided increases of ~ 1.7-fold in sensitivity of diffusion experiments compared with the original XSTE experiment.

To investigate the effect of protein structure on PLRE-induced sensitivity enhancements, the above experiments were repeated using the IDP α-synuclein. SOFAST-HMQC sensitivity enhancements of 1.5-fold were observed for α-synuclein (in 80 mM NiDO2A) (Fig. 1e), and mean residue-specific increases of 1.36 ± 0.40 in 40 mM NiDO2A (Fig. S2). These data indicate that the magnitude of the solvent PRE effect is slightly greater to that observed for ddFLN5 (Fig. 1b), which we suggest is due to the greater solvent accessibility of residues in the IDP allowing a closer approach of the paramagnetic agent (Eqs. 3, 4).

In diffusion measurements of α-synuclein, addition of 20 mM NiDO2A resulted in a small increase of 10 % in XSTE sensitivity, but at higher NiDO2A concentrations this enhancement was found to decrease (Fig. 1f). This is due to the recovery time of 1 s used in XSTE experiments being unnecessarily long given the faster ^1^H longitudinal relaxation rates observed for α-synuclein (Table 1), resulting in a decrease in sensitivity due to enhanced transverse relaxation. In contrast, in the absence of NiDO2A 2.6-fold greater sensitivity was observed using SORDID compared to XSTE, and this increased to 4.8-fold with 80 mM NiDO2A at optimal diffusion delays. Once again, the shorter recovery times in SORDID experiments which are coupled with the diffusion delay are highly beneficial, so higher $R_{1}^{\text{H}}$ induced by higher concentrations of NiDO2A require shorter *T*_rec_, and subsequently result in greater sensitivity. Although the additional hard pulses in the SORDID experiment during $R_{1}^{\text{H}}$ recovery reduce selectivity of excitation (as previously discussed), highly labile, solvent-exposed amide protons allow additional gains in ^1^H longitudinal relaxation, and therefore sensitivity, from increased amide/water proton chemical exchange. These two opposing effects result in fitted $R_{1}^{\text{H}}$ rates which agree well with those measured by a non-selective pulse for globular ddFLN5, but faster fitted $R_{1}^{\text{H}}$ rates than those observed using a hard pulse for disordered α-synuclein due to greater proton exchange. This results in excellent agreement with those measured directly using selective inversion pulses for α-synuclein (Table 1). These effects are also reflected in the fitted global scaling factors for the XSTE and SORDID experiments, where for ddFLN5 good agreement was found between these two values, but a larger scaling factor (and therefore additional sensitivity) was found for SORDID than XSTE experiments using α-synuclein.

As large improvements in the sensitivity of NMR diffusion measurements were readily achieved for isolated proteins using the PLRE agent, we next explored its utility in time-limited measurements of large and dilute macromolecular complexes where increases in sensitivity are a more essential requirement (see “Introduction”). We selected the ~2.4 MDa 70S ribosome particle, the ubiquitous macromolecular machine for protein biosynthesis, as a test case, for despite the large molecular weight sharp resonances can be observed for flexible regions of the L7/L12 stalk complex (Christodoulou et al. 2004; Mulder et al. 2004). The magnitude of the PLRE effect was investigated using a NiDO2A concentration of 40 mM as this was the concentration at which optimum sensitivity enhancement had been observed for both diffusion and 2D NMR measurements of the globular ddFLN5 protein.

**Fig. 2:**
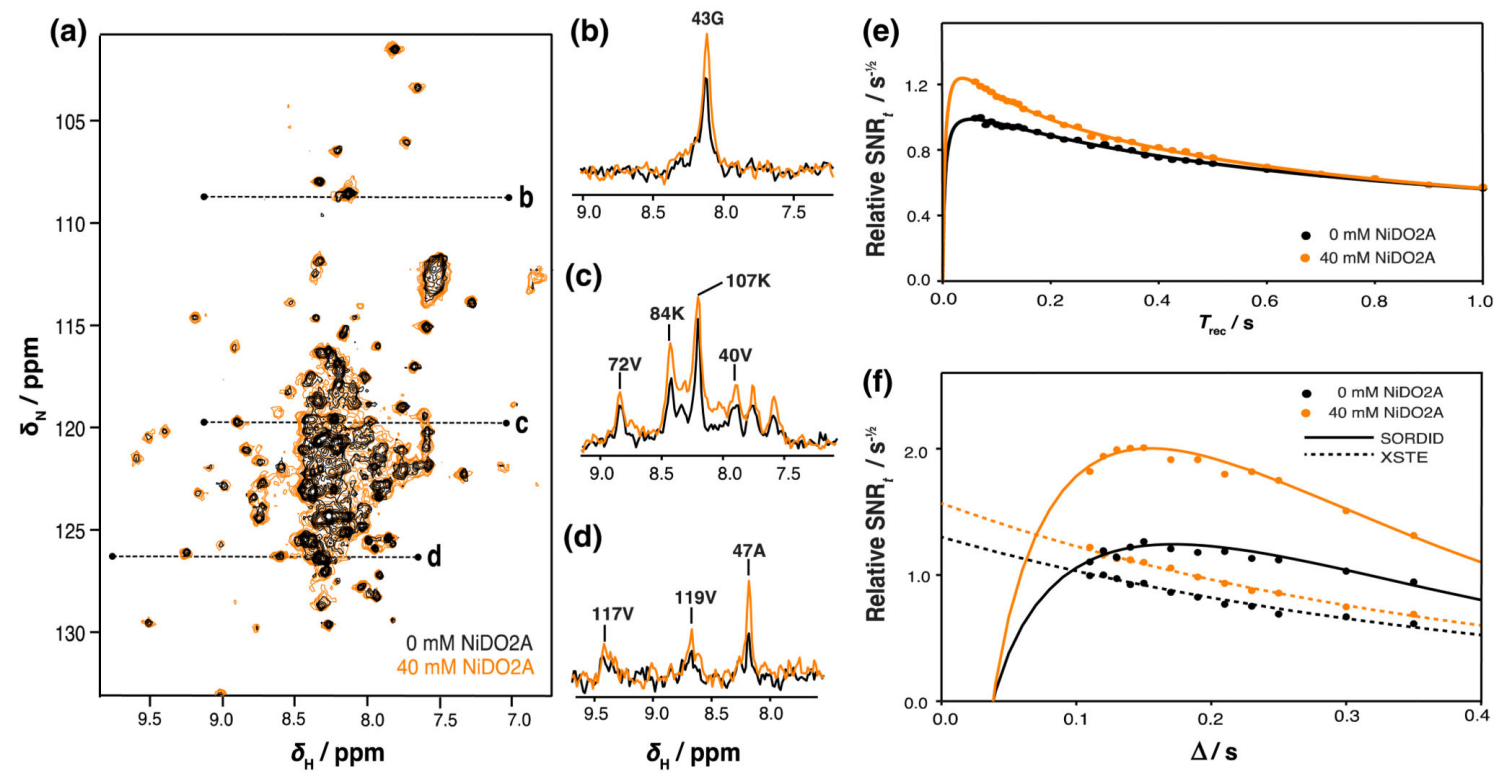
The effect of paramagnetic longitudinal relaxation enhancement on the sensitivity of NMR measurements of *E. coli* 70S ribosomes. **a** Overlay of 2D ^1^H–^15^N SOFAST-HMQC spectra in the presence and absence of 40 mM NiDO2A PLRE agent. *Dashed lines* indicate locations of one-dimensional slices as shown in **b, c, d** and labeled with known assignments from the L7/L12 stalk complex. The relative sensitivity of **e** 1D ^1^H–^15^N SOFAST-HMQC experiments as a function of the recovery delay *T*_rec_, and of **f** XSTE and SORDID diffusion experiments as a function of the diffusion delay Δ. Data are fitted to their theoretical sensitivity expressions (Eqs. 8, 9)

By recording ^1^H–^15^N SOFAST-HMQC spectra of the ribosomes (Fig. 2a) we obtained well-resolved resonances deriving mainly from the L7/L12 ribosomal stalk region (Christodoulou et al. 2004). No chemical shift changes were observed on addition of 40 mM NiDO2A, demonstrating no significant interaction of the ribosome with NiDO2A at this concentration. However, there was an increase in the intensity of the amide envelope of 20 % (Fig. 2e), and in 2D SOFAST-HMQC spectra we found residue-specific sensitivity increases of 1.44 ± 0.25 (SD) (Fig. S2) which allowed some very weak resonances to be better discerned (Fig. 2b-d). Despite some additional line broadening induced by increases in transverse relaxation rates on addition of 40 mM NiDO2A (Table 1), resonances remained in general well-resolved in the 2D spectrum.

The sensitivity of XSTE and SORDID diffusion measurements was investigated in a similar manner as for isolated α-synuclein and ddFLN5 proteins, by measuring the sensitivity enhancements across a range of diffusion delays in the absence and presence of 40 mM NiDO2A (Fig. 2f). We found that the combined use of SORDID and NiDO2A provided a twofold increase in sensitivity: significantly greater than by either method alone, which showed sensitivity gains of approximately 10 and 20 % respectively. Upon fitting of the diffusion data, good agreement was once again found between the directly measured and fitted relaxation rates (Tables 2, 3). Importantly, monitoring of the diffusion coefficient of the ribosomes over time revealed no differences in the integrity or stability of ribosomes arising from the addition of NiDO2A: sample lifetimes observed were between 29 and 47 h (Fig. S3), which we find is typical of ribosome preparations.

**Fig. 3:**
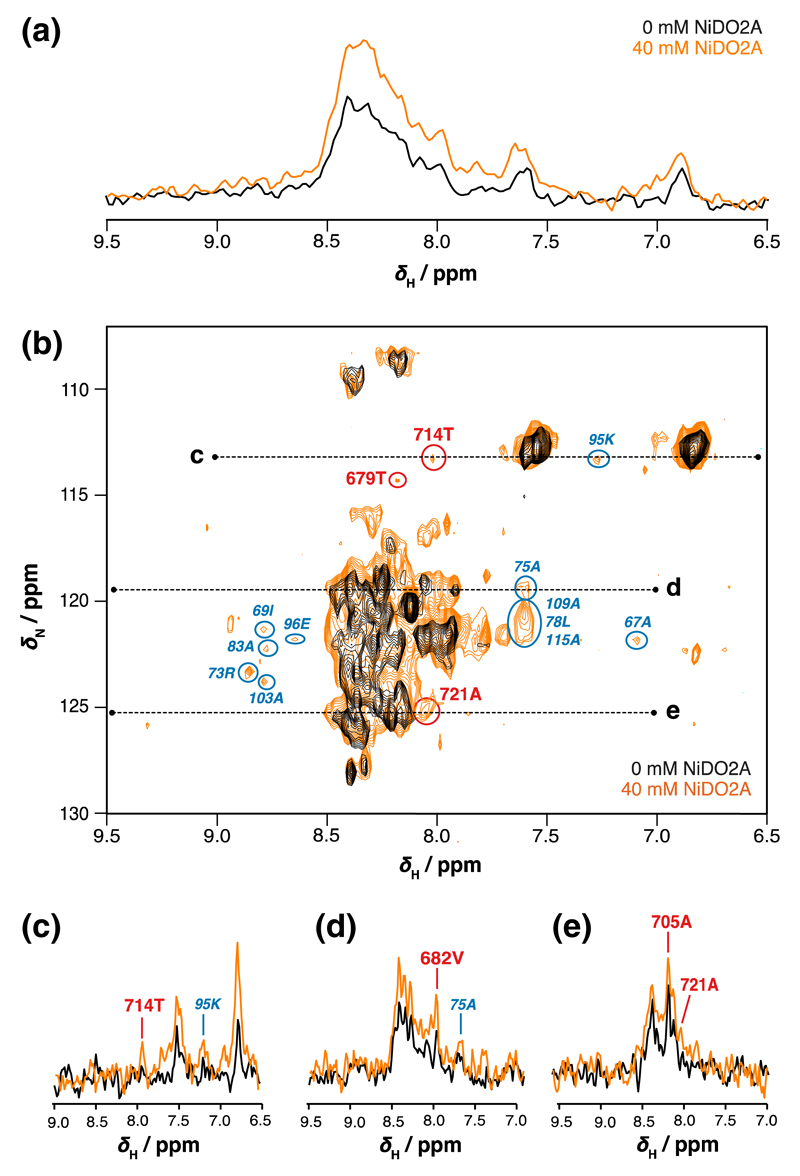
The effect of paramagnetic longitudinal relaxation enhancement on the sensitivity of SOFAST-HMQC experiments of a ddFLN5 RNC. Overlay of **a** 1D ^1^H–^15^N SOFAST-HMQC spectra and of **b** 2D ^1^H–^15^N SOFAST-HMQC spectra (of 30 min experimental time each) in the presence and absence of 40 mM NiDO2A. Additional resonances are observable in the PLRE-induced spectrum and all can be assigned to the nascent chain or the L7/L12 stalk region of background-labeled ribosomes. Those that can be assigned unambiguously to either region are *circled* in *red* or *blue* respectively, and labeled with their known assignments. *Dashed lines* indicate locations of one-dimensional slices as shown in **c, d, e** and labeled with known and unambiguous assignments of unfolded ddFLN5 or L7/12

As previously discussed, there are significant challenges in working with RNCs by NMR spectroscopy due primarily to the low concentration and short lifetimes of these samples. We were therefore motivated to explore the benefits of paramagnetic longitudinal relaxation-induced sensitivity gains as applied to these complex species. We extended our experiments to a ribosome–bound nascent chain of ddFLN5, linked with a 31-residue sequence derived from ddFLN6 and the SecM motif to cause translational stalling of the ribosome (Evans et al. 2005), and in which the selectively ^15^N-labelled nascent chain adopts an unfolded conformation (Cabrita et al. in preparation). We acquired ^1^H–^15^N SOFAST-HMQC correlation spectra (Fig. 3a, b), and observed an enhancement in the amide proton signals of ~ 85 % upon addition of 40 mM NiDO2A (Fig. 3a). This large increase in sensitivity is comparable to that obtained for α-synuclein (Fig. 1), consistent with the disordered nature of the nascent chain. Furthermore, as a result of such large sensitivity enhancements, a significantly greater number of well-resolved cross-peaks become observable within the same experimental time (30 min). At least three of these resonances could be unambiguously assigned to the attached nascent chain, while additional resonances could be attributed to background labeling of the L7/L12 stalk region (Fig. 3b). Importantly, resonances deriving from the nascent chain did not show chemical shift changes in the presence of 40 mM NiDO2A, and examination of one-dimensional slices showed that the signal enhancements far outweigh the smaller increases in line broadening (Fig. 3c-e).

**Fig. 4:**
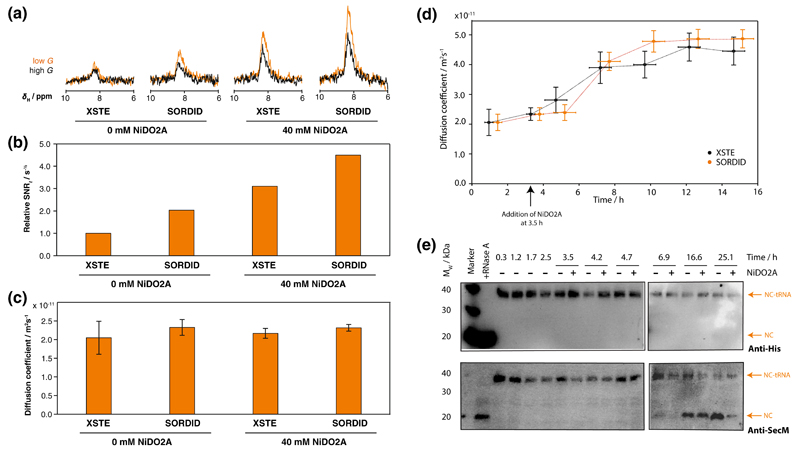
Comparison of NMR diffusion measurements of a ddFLN5 RNC, and monitoring its integrity using both biochemical and NMR analysis. **a** XSTE (Δ = 100 ms; *G* = 5 %, 95 % *G*_max_) and SORDID (Δ = 190 ms; *G* = 10.4 %, 69.5 % *G*_max_) spectra acquired within 30 min each, in the presence and absence of 40 mM NiDO2A. **b** Relative sensitivity for each diffusion experiment. **c** Diffusion coefficients measured by each diffusion experiment. **d** Diffusion coefficients of the RNC sample measured by sets of XSTE and SORDID experiments to examine the integrity and stability of the RNC. **e** Western blot analysis to assess the effect of 40 mM NiDO2A on the RNC integrity, detected using anti-His (*upper*) and anti-SecM (*lower*) antibodies. Upshifted bands (~40 kDa) correspond to the tRNA-bound form of the RNC in which the nascent chain is attached to the ribosome, with release of the nascent chain (and tRNA) monitored by the band at a lower molecular weight of ~23 kDa

XSTE and SORDID diffusion spectra of the RNC were acquired using identical experimental times (30 min) (Fig. 4a). Longitudinal relaxation optimization, achieved by using SORDID or introducing 40 mM NiDO2A into the sample, resulted in 2- and 3.1-fold enhancements in sensitivity respectively, compared to XSTE experiments in the absence of PRE agent (Fig. 4b). However, even greater sensitivity gains were achieved when both methods were combined, and 4.5-fold enhancement in sensitivity was observed. Importantly, all four measurements yielded identical diffusion coefficients (Fig. 4c), corresponding to a diffusion coefficient of (2.22 ± 0.13) × 10^–11^ m^2^ s^–1^ and therefore indicating that the observed resonances were indeed ribosome associated.

The integrity of the RNC sample was assessed both before and after the addition of NiDO2A to the sample, and then over time, by repeatedly acquiring sets of XSTE and SORDID experiments (Fig. 4d). Both the XSTE and SORDID measurements showed a constant diffusion coefficient over the first ~5 h of data acquisition (during which the previously discussed experiments were completed), before increases in the diffusion coefficient were observed after ~7 h, indicating degradation of the sample and release of the nascent chain from the ribosome. These measurements were complemented by biochemical analysis of identical samples, incubated with and without 40 mM NiDO2A, in parallel with the NMR acquisition period. Aliquots of these samples were taken at intervals, and analysed by western blotting to assess the integrity of the nascent polypeptide. Both anti-His and anti-SecM western blots showed a decrease in intensity of the tRNA-bound form of the NC (~40 kDa) and the subsequent appearance of a ~23 kDa species at ~7 h, which corresponds to the released nascent chain; no difference in stability was observed between samples incubated with and without NiDO2A (Fig. 4e). These observations are consistent with the time-course of the NMR diffusion measurements. Taken together with the SOFAST-HMQC data, we conclude that there is no evidence of interaction of NiDO2A with ribosomes or RNCs, and no indication of any effect on the nascent chain or ribosomal particle stability.

## Discussion

Advances in improving the sensitivity and resolution of NMR spectroscopy, such as the development of TROSY methodologies, advanced isotopic labeling strategies, and the greater availability of high-field spectrometers equipped with cryogenic probes, have allowed the application of the technique to increasingly larger and more complex systems. The observation of such large and often unstable macromolecular assemblies requires both continuing improvements in NMR sensitivity and a means to monitor the sample integrity. In this work, we have investigated the application of the paramagnetic longitudinal relaxation enhancement (PLRE) effect in improving the sensitivity of heteronuclear NMR diffusion measurements that are essential in probing the sample stability of ribosomal particles including RNCs. Significant improvements were observed by using the PLRE agent NiDO2A (Cai et al. 2006), particularly in combination with the longitudinal relaxation-optimized SORDID experiment (Augustyniak et al. 2012), for which synergistic enhancements of up to 1.7- and 4.8-fold in sensitivity were observed for globular and disordered proteins respectively, relative to conventional XSTE experiments (Ferrage et al. 2003), without adverse effect on the storage of magnetization on N_*z*_ during the diffusion period and with only minimal line broadening of resonances. Similarly, for an RNC in an unfolded conformation a 4.5-fold increase in diffusion NMR sensitivity was observed. These gains are in addition to improvements in sensitivity of 2D SOFAST-HMQC measurements of 20–85 %, as previously reported for α-synuclein (Theillet et al. 2011).

Paramagnetic longitudinal relaxation enhancement occurs to different extents both between globular and disordered proteins and throughout protein sequences, and this may be rationalized by a combination of two effects. Firstly, the greater solvent accessibility of amides in disordered and less structured regions increases the proximity of the PLRE agent, increasing the strength of dipolar interactions (distance of closest approach in Eqs. 3, 4). Secondly, the PLRE agent will also accelerate the longitudinal relaxation of water protons, and their exchange with amide protons in IDPs will therefore also lead to an increase in the effective $R_{1}^{\text{H}}$ rates (Gil et al. 2013; Cai et al. 2006).

Thus far we have focused our discussion on the generally favourable paramagnetic enhancement of longitudinal relaxation. However, this is inevitably also associated with the enhancement of transverse relaxation (Eqs. 3, 4), and although choices of fast relaxing paramagnetic species such as Ni^(II)^ or Fe^(III)^ can reduce this effect significantly (see “Introduction”) some additional line broadening is unavoidable. For example, the observed doubling of the average proton *R*_2_ rates of α-synuclein upon addition 80 mM NiDO2A results in a twofold increase in proton line widths. However, for many applications, this is considerably outweighed by more than fourfold increases in both longitudinal relaxation rates and sensitivity enhancements, and indeed SOFAST-HMQC spectra acquired after addition of the PLRE agent continued to show well-resolved resonances with few additional overlapped peaks. Clearly, therefore, the optimal concentration of PLRE agent must be judged on a case-by-case basis based on the acceptable compromise between sensitivity and resolution. We note that the addition of soluble PLRE agents also increases the line width of water ^1^H nuclei, which can reduce the effectiveness of water suppression. However, we found that this could be largely alleviated by the introduction of phase cycling to select heteronuclear coherence transfer pathways within the SORDID experiment (Fig. S1), and processing spectra with standard solvent suppression filters and baseline correction (see “Experimental section”).

The gains in sensitivity obtained by paramagnetic longitudinal relaxation enhancement have here been shown to be particularly advantageous to time-limited investigations of large, complex macromolecular assemblies such as RNCs. In NMR diffusion experiments, the additional sensitivity can be exploited to reduce the uncertainty in diffusion coefficients measured within the same experimental time (Fig. 4c). However, given the limited lifetime of an intact RNC, the additional sensitivity can alternatively be used to substantially decrease the required measurement time and more rapidly assess the sample stability. Given the 4.5-fold increase in sensitivity we observe here, the same quality of spectra acquired by XSTE experiments in 30 min may be obtained in less than 1.5 min by combined use of NiDO2A and SORDID. As sample integrity and lifetimes are not compromised on addition of the PLRE agent, the additional available time may therefore be used for longer acquisition of other (for example, 2D) NMR experiments. This can allow higher resolution spectra to be obtained, as previously demonstrated (Theillet et al. 2011). Alternatively, longer acquisition times may be used to increase the sensitivity of existing experiments. For example, we have typically acquired 2D and diffusion measurements for equal periods. By increasing the time allocated to 2D measurements, we estimate the net sensitivity increase (in combination with PLRE-induced gains) to be approximately 2.6-fold. We expect this will greatly facilitate future measurements of RNCs and similar challenging biological systems.

## Electronic supplementary material

The online version of this article (doi:10.1007/s10858-015-9968-x) contains supplementary material, which is available to authorized users.

Supplementary Material

## Figures and Tables

**Table 1:** Longitudinal relaxation rates of ddFLN5 (above) and α-synuclein (below) as a function of NiDO2A concentration, from direct measurements using inversion-recovery experiments with non-selective or band-selective pulses, and determined from fitting of XSTE and SORDID experimental data

[NiDO2A]/mM	*R*_1_ (^15^N)/s^–1^			*R*_1_ (^1^H)/s^–1^			
	Observed	XSTE fit	SORDID fit	Observed hard	Observed amide-selective	XSTE fit	SORDID fit
ddFLN5^a^							
0	2.00 ± 0.26	1.73 ± 0.18	2.31 ± 0.13	0.77 ± 0.03	5.49 ± 1.31	0.85 ± 0.27	1.26 ± 0.19
20	1.98 ± 0.26	1.91 ± 0.19	2.62 ± 0.14	1.24 ± 0.06	6.15 ± 1.10	1.76 ± 2.81	1.62 ± 0.25
40	1.99 ± 0.28	1.70 ± 0.15	2.82 ± 0.15	1.75 ± 0.05	6.88 ± 1.12	2.26 ± 3.15	1.98 ± 0.31
60	2.02 ± 0.26	1.60 ± 0.28	2.61 ± 0.16	2.57 ± 0.08	7.93 ± 0.95	8.69 ± 9.27	2.31 ± 0.36
α-Synuclein^b^							
0	2.57 ± 0.29	1.59 ± 0.17	1.73 ± 0.07	1.28 ± 0.09	4.43 ± 0.24	2.49 ± 0.51	3.03 ± 0.15
20	2.41 ± 0.30	1.44 ± 0.17	1.94 ± 0.10	3.70 ± 0.07	6.85 ± 0.22	7.82 ± 2.83	5.75 ± 0.32
40	2.40 ± 0.31	1.62 ± 0.14	1.77 ± 0.14	5.77 ± 0.09	8.90 ± 0.24	8.57 ± 5.17	8.44 ± 0.51
60	2.33 ± 0.30	1.79 ± 0.12	1.68 ± 0.18	7.79 ± 0.08	10.89 ± 0.26	10.40 ± 7.74	11.04 ± 0.71
80	2.32 ± 0.31	2.00 ± 0.16	1.50 ± 0.23	9.74 ± 0.14	13.00 ± 0.33	14.20 ± 8.14	14.11 ± 1.00

a Scaling coefficients: *A*_XSTE_ = 3.52 ± 0.76 (Eq. 8); *A*_SORDID_ = 4.96 ± 0.69 (Eq. 9)

b Scaling coefficients: *A*_XSTE_ = 1.54 ± 0.27 (Eq. 8); *A*_SORDID_ = 3.55 ± 0.18 (Eq. 9)

**Table 2:** Measured ^1^H and ^15^N transverse relaxation rates of α-synuclein, ddFLN5, and *E. coli* 70S ribosomes as a function of NiDO2A concentration

[NiDO2A]/mM	*R*_2_ (^15^N)/s^–1^			*R*_2_ (^1^H)/s^–1^		
	α-Synuclein	ddFLN5	70S ribosomes	α-Synuclein	ddFLN5	70S ribosomes
0	3.62 ± 0.11	10.19 ± 0.19	6.92 ± 2.06	11.01 ± 0.16	24.61 ± 2.55	32.24 ± 6.24
20	3.74 ± 0.37	10.27 ± 0.20	−	14.91 ± 0.44	27.46 ± 1.30	−
40	3.81 ± 0.07	10.45 ± 0.11	8.03 ± 1.77	18.43 ± 1.51	29.16 ± 1.99	45.71 ± 5.86
60	3.89 ± 0.05	10.59 ± 0.41	−	21.43 ± 0.53	34.14 ± 2.20	−
80	3.94 ± 0.05	−	−	23.91 ± 0.66	−	−

**Table 3:** Longitudinal relaxation rates of *E. coli* 70S ribosomes as a function of NiDO2A concentration, from direct measurements using inversion-recovery experiments with non-selective or band-selective pulses, and determined from fitting of XSTE and SORDID experimental data

[NiDO2A]/mM	*R*_1_ (^15^N)/s^−1^			*R*_1_ (^1^H)/s^−1^			
	Observed	XSTE fit	SORDID fit	Observed hard	Observed amide-selective	XSTE fit	SORDID fit
0	2.69 ± 0.44	1.87 ± 0.16	3.55 ± 0.22	1.09 ± 0.39	11.02 ± 3.32	1.63 ± 0.26	1.82 ± 0.45
40	2.40 ± 0.71	1.99 ± 0.13	3.78 ± 0.30	1.70 ± 0.39	13.34 ± 3.88	1.77 ± 0.31	2.92 ± 0.76

Scaling coefficients: *A*_XSTE_ = 2.48 ± 0.24 (Eq. 8); *A*_SORDID_ = 4.78 ± 0.72 (Eq. 9)

## References

[R1] Augustyniak R, Ferrage F, Paquin R, Lequin O, Bodenhausen G (2011). J Biomol NMR.

[R2] Augustyniak R, Ferrage F, Damblon C, Bodenhausen G, Pelupessy P (2012). Chem Commun (Camb).

[R3] Baldwin AJ, Anthony-Cahill SJ, Knowles TPJ, Lippens G, Christo-doulou J, Barker PD, Dobson CM (2008). Angew Chem Int Ed Engl.

[R4] Bernini A, Venditti V, Spiga O, Niccolai N (2009). Prog Nucl Magn Reson Spectrosc.

[R5] Bertini I, Luchinat C, Parigi G (2001). Solution NMR of paramagnetic molecules: applications to metallobiomolecules and models.

[R6] Cabrita LD, Hsu S-TD, Launay H, Dobson CM, Christodoulou J (2009). Proc Natl Acad Sci USA.

[R7] Cai S, Seu C, Kovacs Z, Sherry AD, Chen Y (2006). J Am Chem Soc.

[R8] Chang CA, Chen C-Y, Chen H-Y (1999). J Chin Chem Soc.

[R9] Christodoulou J, Larsson G, Fucini P, Connell SR, Pertinhez TA, Hanson CL, Redfield C, Nierhaus KH, Robinson CV, Schleucher J, Dobson CM (2004). Proc Natl Acad Sci USA.

[R10] Chung K-C, Yu H-Y, Ahn S-D (1970). Bull Korean Chem Soc.

[R11] Clore GM, Iwahara J (2009). Chem Rev.

[R12] Delaglio F, Grzesiek S, Vuister GW, Zhu G, Pfeifer J, Bax A (1995). J Biomol NMR.

[R13] Efron B, Tibshirani RJ (1994). An introduction to the bootstrap.

[R14] Eichmann C, Preissler S, Riek R, Deuerling E (2010). Proc Natl Acad Sci USA.

[R15] Eletsky A, Moreira O, Kovacs H, Pervushin K (2003). J Biomol NMR.

[R16] Ernst RR, Anderson WA (1966). Rev Sci Instrum.

[R17] Evans MS, Ugrinov KG, Frese M-A, Clark PL (2005). Nat Methods.

[R18] Fernández C, Wider G (2003). Curr Opin Struct Biol.

[R19] Ferrage F, Zoonens M, Warschawski DE, Popot J-L, Bodenhausen G (2003). J Am Chem Soc.

[R20] Fiaux J, Bertelsen EB, Horwich AL, Wüthrich K (2002). Nature.

[R21] Gil S, Hosek T, Solyom Z, Kuemmerle R, Brutscher B, Pierattelli R, Felli IC (2013). Angew Chem Int Ed.

[R22] Helm L (2006). Prog Nucl Magn Reson Spectrosc.

[R23] Hiller S, Wider G, Etezady-Esfarjani T, Horst R, Wüthrich K (2005). J Biomol NMR.

[R24] Hsu S-TD, Fucini P, Cabrita LD, Launay H, Dobson CM, Christodoulou J (2007). Proc Natl Acad Sci USA.

[R25] Hsu S-TD, Cabrita LD, Fucini P, Dobson CM, Christodoulou J (2009a). J Mol Biol.

[R26] Hsu S-TD, Cabrita LD, Fucini P, Christodoulou J, Dobson CM (2009b). J Am Chem Soc.

[R27] Hyberts SG, Arthanari H, Wagner G (2012). Top Curr Chem.

[R28] Johnson CS (1999). Prog Nucl Magn Reson Spectrosc.

[R29] Kovacs H, Moskau D, Spraul M (2005). Prog Nucl Magn Reson Spectrosc.

[R30] Li C, Wang Y, Pielak GJ (2009). J Phys Chem B.

[R31] Lucas LH, Larive CK (2004). Concepts Magn Reson.

[R32] Madl T, Güttler T, Görlich D, Sattler M (2011). Angew Chem Int Ed Engl.

[R33] Mulder FAA, Bouakaz L, Lundell A, Venkataramana M, Liljas A, Akke M, Sanyal S (2004). Biochemistry.

[R34] Otting G (2010). Annu Rev Biophys.

[R35] Pervushin K, Riek R, Wider G, Wüthrich K (1997). Proc Natl Acad Sci USA.

[R36] Pervushin K, Vögeli B, Eletsky A (2002). J Am Chem Soc.

[R37] Rantaharju J, Mareš J, Vaara J (2014). J Chem Phys.

[R38] Rovnyak D, Hoch JC, Stern AS, Wagner G (2004). J Biomol NMR.

[R39] Schanda P, Kupce E, Brutscher B (2005). J Biomol NMR.

[R40] Sprangers R, Kay LE (2007). Nature.

[R41] Stejskal EO, Tanner JE (1964). J Chem Phys.

[R42] Theillet F-X, Binolfi A, Liokatis S, Verzini S, Selenko P (2011). J Biomol NMR.

[R43] Tugarinov V, Hwang PM, Ollerenshaw JE, Kay LE (2003). J Am Chem Soc.

[R44] Tugarinov V, Kanelis V, Kay LE (2006). Nat Protoc.

[R45] Vranken WF, Boucher W, Stevens TJ, Fogh RH, Pajon A, Llinas M, Ulrich EL, Markley JL, Ionides J, Laue ED (2005). Proteins.

[R46] Waudby CA, Christodoulou J (2012). J Magn Reson.

[R47] Waudby CA, Knowles TPJ, Devlin GL, Skepper JN, Ecroyd H, Carver JA, Welland ME, Christodoulou J, Dobson CM, Meehan S (2010). Biophys J.

[R48] Waudby CA, Mantle MD, Cabrita LD, Gladden LF, Dobson CM, Christodoulou J (2012). J Am Chem Soc.

[R49] Waudby CA, Launay H, Cabrita LD, Christodoulou J (2013). Prog Nucl Magn Reson Spectrosc.

[R50] Wickramasinghe NP, Kotecha M, Samoson A, Past J, Ishii Y (2007). J Magn Reson.

[R51] Wilkins DK, Grimshaw SB, Receveur V, Dobson CM, Jones JA, Smith LJ (1999). Biochemistry.

[R52] Wishart DS, Bigam CG, Yao J, Abildgaard F, Dyson HJ, Oldfield E, Markley JL, Sykes BD (1995). J Biomol NMR.

[R53] Wu D, Chen A, Johnson CS (1995). J Magn Reson.

