# Supplementary material for: Increasing the sensitivity of NMR diffusion measurements by paramagnetic longitudinal relaxation enhancement, with application to ribosome–nascent chain complexes
Source: J Biomol NMR. Author manuscript; Available in PMC 2016 Jun 28. (PMC4924603; doi:10.1007/s10858-015-9968-x)
Supplement: Supplementary Material [file NIHMS68845-supplement-Supplementary_Material.pdf]

Increasing the sensitivity of NMR diffusion measurements by paramagnetic longitudinal relaxation enhancement, with application to ribosome–nascent chain complexes

Sammy H.S. Chan, Christopher A. Waudby, Anaïs Cassaignau, Lisa D. Cabrita, and John Christodoulou*

Institute of Structural and Molecular Biology, University College London and Birkbeck College
Gower St, London WC1E 6BT, United Kingdom

* To whom correspondence should be addressed: j.christodoulou@ucl.ac.uk

Supplementary Material

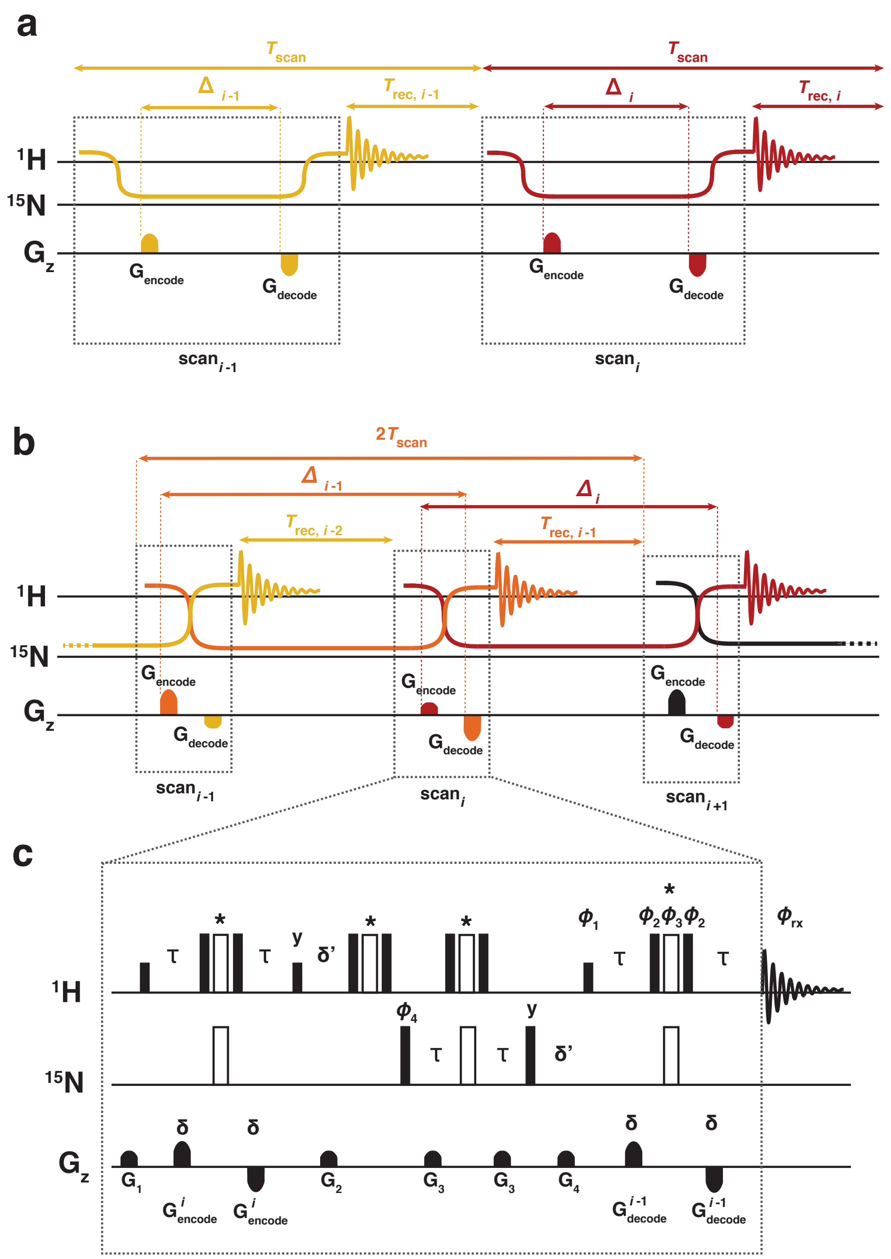

Fig. S1 Schematic (**a**, **b**) and detailed (**c**) pulse sequences for ^{15}N heteronuclear diffusion experiments, adapted from reference [28]. (**a**) In XSTE experiments, after a recovery period T_{rec}, proton magnetization is transferred onto N_z where is it encoded during the diffusion delay Δ and, after decoding, is detected on ^{1}H in one scan. (**b**) In SORDID experiments, the diffusion delay extends over two scans, allowing the recovery time of the previous scan to occur during the diffusion delay of the following scan. (**c**) In the detailed pulse sequence of SORDID, filled and open rectangles represent 90° and 180° pulses, respectively. Hard composite pulses 75°, 285°, 75° are indicated by stars, and amide-selective 90° squared pulses are represented by lower filled rectangles. Unless otherwise mentioned, all pulses are applied along the *x*-axis of the rotating frame. The phase cycling employed was: $\phi_1 = (-x)_8, (-y)_8, x_8, y_8$; $\phi_2 = (34°)_4(124°)_4(214°)_4(304°)_4$; $\phi_3 = (144°)_4(234°)_4(324°)_4(324°)_4$. In addition, with careful consideration of the interleaving scans, the following phase cycling was employed to select heteronuclear coherences: $\phi_4 = x, -x, x, x$; noting that the receiver phase shift $\Delta\varphi_{rx}^{(i)} = \Delta\varphi_4^{(i)}\Delta\varphi_4^{(i-1)}$, the receiver phase was $\phi_{rx} = x, -x, -x, x$. The receiver phase encompassing the two phase cycles was therefore $\phi_{rx} = x, -x, -x, x, -x, x, x, -x, -y, y, y, -y, y, -y, -y, y, -x, x, x, -x, x, -x, -x, x, y, -y, -y, y, -y, y, y, -y$. The INEPT delays were $\tau = -1/4J_{NH} = 2.72$ ms ($J_{NH} \approx -92$ Hz). The encoding and decoding gradients ($G^{i}_{encode} = G^{i}_{decode}$) had variable strengths but constant duration $\delta = 1$ ms.

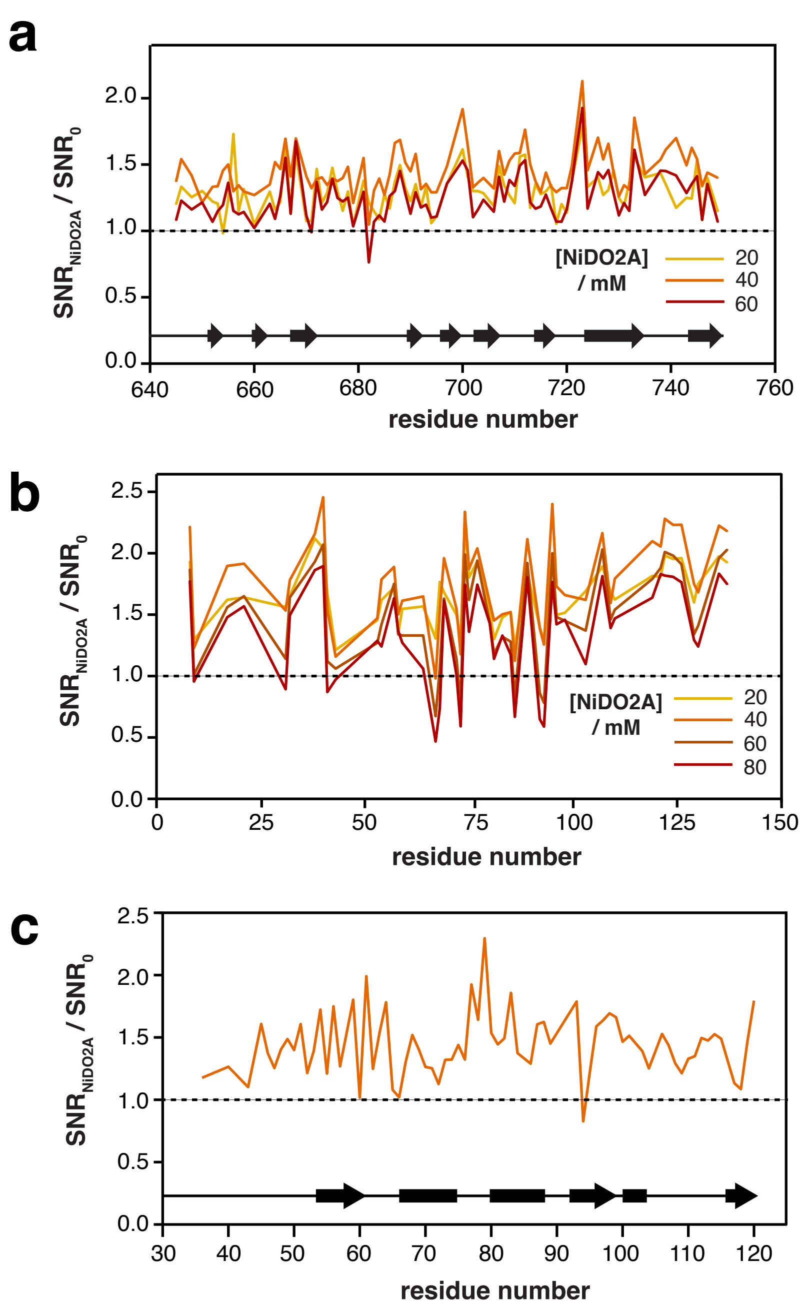

Fig. S2 Residue-specific changes in relative signal-to-noise of 2D SOFAST-HMQC experiments (recovery delay of 50 ms) for (**a**) ddFLN5 upon addition of 20-60 mM NiDO2A (representation of secondary structure is shown below, arrows represent beta strands), mean and standard deviation of 1.30 ± 0.16, 1.45 ± 0.17 and 1.25 ± 0.17 in 20, 40 and 60 mM NiDO2A respectively; (**b**) α-synuclein upon addition of 20-80 mM NiDO2A, mean and standard deviation of 1.66 ± 0.25, 1.77 ± 0.37, 1.49 ± 0.39 and 1.36 ± 0.40 in 20, 40, 60 and 80 mM NiDO2A respectively; and (**c**) the L7/L12 stalk region bound to 70S *E. coli* ribosomes upon addition of 40 mM NiDO2A (representation of secondary structure is shown below, arrows represent beta strands and blocks represent α-helices), mean and standard deviation of 1.44 ± 0.25.

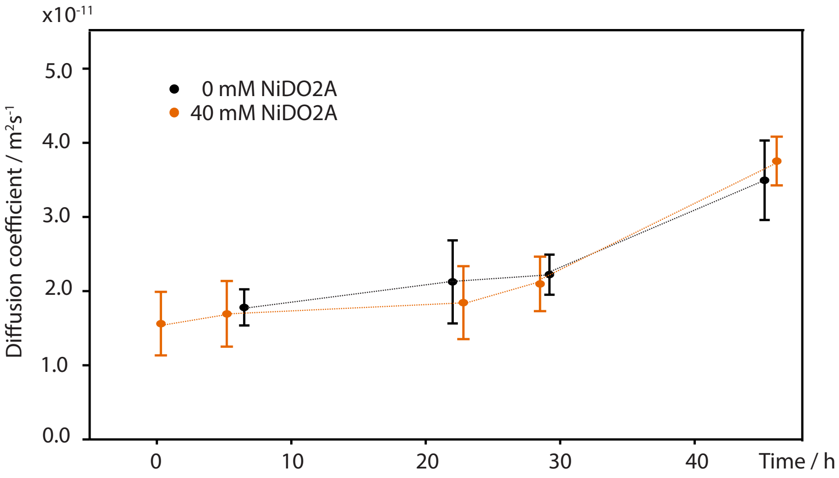

Fig. S3 To assess the integrity of 70S ribosomes during NMR acquisition, and the effect of NiDO2A on the sample stability, its diffusion coefficient was continually monitored using XSTE and SORDID measurements in the presence and absence of 40 mM NiDO2A.
